# Tobacco cessation programs and factors associated with their effectiveness in the Middle East: A systematic review

**DOI:** 10.18332/tid/153972

**Published:** 2022-11-03

**Authors:** Maha R. Al-Qashoti, Retaj Aljassim, Mohamed A. M. Sherbash, Nour W. Z. Alhussaini, Ghadir Fakhri Al-Jayyousi

**Affiliations:** 1 Department of Public Health, College of Health Sciences, QU Health, Qatar University, Doha, Qatar

**Keywords:** tobacco cessation, quit smoking, interventions, Middle East, systematic review

## Abstract

**INTRODUCTION:**

In Middle East countries, the average prevalence of tobacco use is relatively high. This systematic review aimed to explore different tobacco cessation programs provided in the Middle East, identify healthcare professionals providing these programs, and the factors associated with their effectiveness.

**METHODS:**

A systematic review was conducted using an electronic search of PubMed, EMBASE, Cochrane Library, ProQuest, and Web of Science, bibliographic databases between 24 January 2021 and 7 March 2021, to identify all relevant studies. The keywords used were ‘tobacco cessation’ and ‘Middle East’. The review was undertaken applying the Preferred Reporting Items for Systematic Reviews and Meta-Analysis guidelines (PRISMA). Based on the study types, several quality assessment tools including the Cochrane risk of bias tool for randomized controlled trials, MINORS for quasi-experimental studies, NIH for cross-sectional studies, NIH for pre-post studies, and CASP for cohort studies, were used.

**RESULTS:**

Among the 512 studies screened, only 30 were included in this review. Our systematic review identified different cessation methods, with some employing both behavioral change and pharmacological methods, and some utilizing only one method. Physicians are believed to be the most common providers of cessation programs, with only a few other healthcare professionals doing so. The results of this review revealed that several factors are associated with the effectiveness of tobacco cessation programs in the Middle East including individual, interpersonal, community, organizational, policy, and environmental.

**CONCLUSIONS:**

Future research should focus on examining the sociocultural and economic factors that might influence tobacco cessation programs. The included studies were of average to poor quality, highlighting the need to conduct highquality studies. The findings provide evidence to encourage the development of multilevel programs to improve the efficacy of tobacco cessation initiatives in the Middle East.

## INTRODUCTION

Tobacco smoking remains the leading cause of preventable deaths worldwide and poses serious public health concerns^1^. Although the prevalence of tobacco smoking has been steadily declining over the past decade, the number of smokers is still high^2^. Globally, the rate of smoking in men and women has been estimated to be 32.7% and 5.8%, respectively^2^. In Middle East (ME) countries, the average prevalence of tobacco use is relatively high^3^. The Middle East is a geographical and cultural region located primarily in south western Asia and the eastern shores of the Mediterranean Sea. The dominant cultures include Arab, Turkish, and Persian (Iranian). The region contains mainly 15 countries: Turkey, Iran, Egypt, Iraq, Lebanon, Syria, Jordan, Palestine, Kuwait, Saudi Arabia, Bahrain, Qatar, UAE, Oman, and Yemen^4^.

In addition to cigarette smoking, there are several other widespread types of tobacco smoking in the ME, such as waterpipe tobacco smoking (WTS) also known as hookah, shisha, arghila, and narghile, midwakh (Arabic pipe for tobacco smoking), dokha (Arabian tobacco), sniffing, and chewing tobacco. Waterpipe is the second most common tobacco product after cigarettes^5^. This type of smoking is very common in the ME, especially among youth and women, and this could be due to the misconception that it is less harmful than cigarettes and to the less stigma that is associated with it, which makes it an acceptable habit for women and socializing^6^. The prevalence of WTS among university students has been estimated to be 60.7%, 67.7%, and 63.1% in Egypt, Jordan, and Palestine, respectively^7^. Other countries in different parts of the region that reported lower rates of WTS among university students include the Kingdom of Saudi Arabia (KSA) (14.5%)^8^, Qatar (18.1%)^9^, and Iran (17.6%)^10^.

Smoking affects almost all body organs leading to a number of diseases and disabilities. It is also a risk factor for various health issues such as heart disease, cancer, stroke, chronic obstructive pulmonary disease, lung diseases, and diabetes^11^. Tobacco smoking is estimated to be responsible for up to 8 million deaths annually, 7 million deaths are attributed to the use of tobacco^12^. Tobacco smoking is not only detrimental to the smoker’s health, but also to the health of those around them since it has been documented that secondhand smoking results in significant health implications induced by inhaled smoke in the respiratory system^13^. Besides human health, tobacco use may also have an impact on other parameters such as national economies leading to economic destruction of hundreds of billions of dollars worldwide every year^14^.

According to the Centers for Disease Control and Prevention (CDC), smoking cessation services lower the rate of premature deaths and improve quality of life^11^. Smoking cessation can add a decade to the life expectancy of individuals who smoked previously^15^. The 2020 Surgeon General’s Report addressed the findings that emphasize the need to quit smoking to enhance overall health outcomes regardless of age or length of smoking^16^.

There are two major types of tobacco cessation (TC) interventions: non-pharmacological therapies and pharmacotherapies^17^. These two approaches can either be utilized separately or in combination with one another^17^. According to the United States Food and Drug Administration (FDA), non-pharmacological therapies include the use of behavioral treatments, such as counseling, cognitive therapy, and motivational interviewing. It can also include individual, group, and telephone counseling, all of which have been reported to be effective^18^. Pharmacotherapies include seven medications approved by the FDA^16^. These therapies have a high long-term success rate and are used to treat nicotine addiction, when patients are unable to quit or find it difficult to manage due to nicotine^19^. Nicotine replacement therapy (NRT) is a type of pharmacotherapy for smoking cessation. Examples include gums, nasal sprays, patches, inhalers, and lozenges. The other two nicotine-free medications are bupropion and varenicline^18^. Studies have shown that a combination of non-pharmacological therapies and pharmacotherapies is effective for TC^16^. Quitline is a population-focused tobacco cessation strategy that includes both pharmacotherapy and behavioral therapy and has been shown to have many benefits including fewer logistical obstacles and easy access such as minimizing travel costs and other costs associated with quitting smoking^20^.

It is imperative to note that use of theories is recommended for understanding the factors that contribute to smoking behavior and assist in developing smoking cessation interventions^21^. Toward this, the trans-theoretical model (TTM) is a helpful tool for assessing patients’ readiness to quit tobacco use by adopting both pharmacological and non-pharmacological therapies. TTM is composed of five main stages to motivate patients to quit: precontemplation (no plan to change within the coming 6 months), contemplation (thinking of quitting within the coming 6 months), preparation (planning to quit for the next 30 days), action (quitting successfully for less than 6 months), and maintenance (quitting successfully for 6 months or more)^17^.

According to the National Cancer Institute at the National Institutes of Health, smoking cessation is to quit smoking^22^. Smoking cessation outcomes are measured in two ways, self-report measures and biochemical validation tests. Based on consensus reached by experts, the six and/or twelve months continuous abstinence, the seven-day point-prevalence abstinence, and the number of cigarettes smoked after seven days are all important self-report measures for cessation, in addition to the biochemical test for levels of cotinine, which is a metabolite of nicotine that can be measured through urine samples^23^. Continuous abstinence is defined as not smoking for several months after a quitting attempt, while point-prevalence abstinence is not smoking on the day of follow-up or a couple days before.^24^

Several factors contribute to the success of TC programs. These are classified as multilevel factors, with individual and environmental influences predominating. Individual characteristics include smoking duration, nicotine dependence, nicotine withdrawal syndrome severity, history of failed quitting attempts, genetic factors, low self-efficacy, fear of weight gain, stress, negative mood, and depression. Social factors, tobacco marketing, and cue reactivity, are examples of environmental factors that may contribute to the effectiveness of the programs^25^. To help countries lower tobacco demand and supply, the World Health Organization (WHO) Framework Convention on Tobacco Control (FCTC) was established^26^. In all, 182 nations had ratified the FCTC as of 2020 and committed themselves to putting the suggested tobacco control measures into effect. All Middle East countries have signed the FCTC^26^. However, only a few of the countries have implemented or consistently enforced FCTC policies^6^.

Clinicians and different healthcare providers (HCPs) must be involved in TC programs, especially those who have direct contact with patients because they are in a better position to help patients quit smoking than others^18^. Furthermore, research has shown that it is crucial to train healthcare providers involved in TC programs to improve the outcomes of these programs^27^. Decision makers should also be involved, where the integration of clinicians’ efforts with healthcare systems, insurance companies, and investors, provides a chance to raise the rate of tobacco dependence treatments, quitting rates, and successful TC^18^.

There has been a surge in the use of validated and effective TC programs in the ME^28^, which has led to effective outcomes toward quitting smoking; however, no reviews have examined the specific programs used in the region. Furthermore, it is crucial to investigate the involvement of HCPs in tobacco cessation programs. This is because a study revealed a lack of specialization and training in this area in the ME^29^. With gaps implying a deeper and profound shortcoming in this specific area, the present systematic review aims to explore the different TC programs available in the ME and identify the factors associated with the effectiveness of these programs.

## METHODS

### Study design

A systematic review was undertaken using the Preferred Reporting Items for Systematic Reviews and Meta-Analysis guidelines (PRISMA)^30^. Meta-analysis was not considered in this study, since results from different studies could not be combined.

### Search strategy

An electronic search of PubMed, Embase, Cochrane, ProQuest, and Web of Science bibliographic databases was conducted between 24 January 2021 and 7 March 2021, to identify all relevant studies published in peer-reviewed journals from 2000–2021. The keywords ‘Tobacco Cessation’ and ‘Middle East’ were combined using Boolean operators (AND and OR). The combination of keywords and Medical Subject Headings (MeSH) terms used in the systematic search was: [tobacco cessation OR smoking cessation OR quitting smoking OR stopping smoking OR smoking absence] AND [Middle East OR Egypt OR Palestine OR Jordan OR Lebanon OR Syria OR Qatar OR Oman OR Bahrain OR United Arab Emirates OR Saudi Arabia OR Kuwait OR Yemen OR Iran OR Turkey OR Iraq] (Table 1).

**Table 1:** Search combinations of keywords and MeSH terms

MeSH terms for TC	MeSH terms for ME
Tobacco cessation OR smoking cessation OR quitting smoking OR stopping smoking OR smoking abstinence	Bahrain OR Egypt OR Iran OR Iraq OR Jordan OR Kuwait OR Lebanon OR Oman OR Palestine OR Qatar OR Saudi Arabia OR the Syrian Arab Republic OR Turkey OR the United Arab Emirates OR Yemen

MeSH: Medical Subject Headings. TC: tobacco cessation. ME: Middle East.

### Eligibility criteria

Studies that met the following criteria were included:

1. Factors: investigating factors associated with the effectiveness of the TC program (experimental or observational);
2. Setting: conducted in the ME region based on the World Atlas definition for the region;
3. Outcome: including results of assessment of the (e.g. quitting rate); and
4. Language: published in the English language.

Studies with the following parameters were excluded:

1. Those that did not address any TC program;
2. Those that were narrative reports that assessed opinions or perceptions regarding tobacco cessation methods;
3. Those published as conference abstracts; and 4. Those of which full-texts could not be accessed.

### Screening and data extraction

The titles and abstracts of the studies were identified and screened for relevance following the predetermined inclusion criteria (articles focusing on tobacco cessation programs and factors associated with their effectiveness), the first author uploaded the selected studies on the endnote program and removed duplicates, and if deemed eligible, only one article with comprehensive information was included in the review. During the screening process, the first two authors and the corresponding author would meet to resolve any conflict and reach an agreement. Next, a word document spreadsheet was created with the required information to be extracted. The full texts of relevant studies were accessed, and their eligibility for inclusion was evaluated.

The first two authors performed data extraction, independently. The studies were cross-checked and discussed by the two authors to resolve any disagreement. Basic information, such as author name, country, year of publication, study type, cessation methods, setting of the programs, program providers, aim of the studies, outcomes reported, and factors associated with the effectiveness of the TC programs, were retrieved from each study. All processes were performed independently; if issues arose, a discussion with the corresponding author was conducted to resolve any conflicts.

### Quality assessment

The methodological quality and risk of bias of the included studies were independently assessed by two authors (MA and RA). Different tools including the Cochrane risk of bias tool for randomized controlled trials (RCTs)^31^, methodological item for non-randomized studies (MINORS) for quasi-experimental studies^32^, the National Heart Lung and Blood Institute (NIH) quality assessment tool for cross-sectional studies^33^, NIH quality assessment tool for pre-post-studies^33^, and critical appraisal skills program (CASP) for cohort studies^34^ specific to study design were used.

## RESULTS

### Study selection

**Figure 1:**
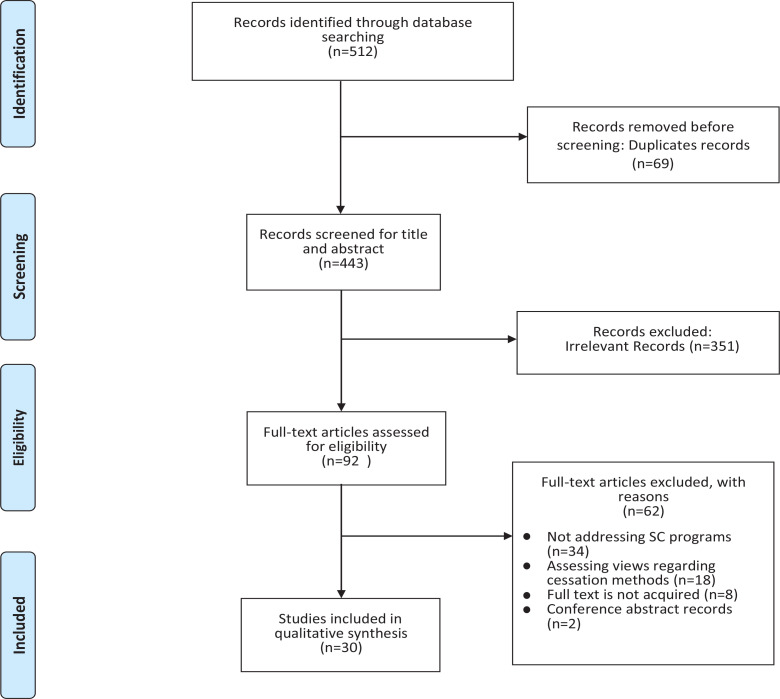
PRISMA flow diagram for study selection

The search strategy resulted in a total of 512 studies. Among these, 69 duplicates and 351 irrelevant studies were excluded. Next, of the 92 full-text assessed studies, 30 met the inclusion criteria and were included in our systematic review. A flow chart depicting the eligible studies and the reasons for exclusion of full-text articles is presented in Figure 1. The authors of some publications and conference papers were personally contacted to obtain full-text articles and information on whether the studies were peer-reviewed; some articles were delivered, and when no response was received from the authors, the study was excluded.

### Study characteristics

The characteristics of the studies included in this systematic review are summarized in Table 2. We identified studies across multiple countries: Turkey (n=13), Iran (n=11), and Jordan (n=2), and one study was conducted in each of Syria, Qatar, Bahrain, and Lebanon. The included studies employed different study designs, with the majority employing a cohort study (n=12), followed by RCTs (n=9), cross-sectional studies (n=4), pre-post studies (n=3), and quasi-experimental studies (n=2). Over 50% of the studies (n=18) used both qualitative and quantitative methods for data compilation. Further, most of the included studies (79%) were conducted in TC clinics, hospitals, and universities. The sample size of the cohort studies that were published between 2007 and 2020 ranged from 100 to 34154, and 41400 were tobacco smokers. Twenty-five of the included studies reported males to be the majority of their participants, and only two studies reported almost equal representation of both genders.

**Table 2:** Description and characteristics of the selected studies

Authors Country Year	Type of study	Sample size	Age of TCCs patients (years) Mean ± SD	Gender	Cessation method – behavioral change, non-pharmacological	Cessation method – pharmacotherapy	Setting	Program providers
Yilmaz et al.^42^ Turkey 2006	Randomized controlled trial	363	Majority <16	100% females	Education and written documents about how to quit smoking	NA	Tertiary referral center	Nurses, and other personnel
Ybarra et al.^35^ Turkey 2012	Randomized controlled trial	150	36.1 ± 9.5	39.1% females, 60.9% males	Telephone-based counseling	NA	Local shopping malls, advertisements in local newspapers, and on Hacettepe University campus	Resident assistant
Heydari et al.^44^ Iran 2012	Randomized controlled trial	272	42.43 ± 13.4	41.2% females, 58.8% males	Brief phone behavior therapy counseling and education	Varenicline treatment vs nicotine replacement medications	Tobacco cessation clinics in the Tobacco Prevention and Control Research Centre	Physicians and nurses
Ward et al.^57^ Syria 2013	Randomized controlled trial	269	Placebo group: 40.0 ± 11.4 Nicotine patch group: 39.9 ± 11.4	Intervention group: 24.6% females, 75.4% males Control group: 18.5% females, 81.5% males	Face -to-face behavioral cessation counseling, and telephone support	Nicotine patches (active, placebo)	Primary care clinics	Physicians
Koyun and Eroğlu^60^ Turkey 2016	Randomized controlled trial	72	Intervention group: 33.3 ± 7.7 Control: 34.0 ± 7.7	100% females	TTM-based training, TTM-based individual counseling, and TTMbased self-help material	NA	Family health centers	Researchers
Aryanpur et al.^40^ Iran 2016	Randomized clinical trial	210 newly diagnosed pulmonary TB patients with smoking habit	Majority ≥18	Intervention group 1: 9.7% females, 90.3% males Intervention group 2: 10% females, 90% males Control group: 9.7% females, 90.2% males	Brief advice and individualized counseling session of quitting behavioral therapy	Treatment with slow- release bupropion	Six selected clinics in Tehran	Provided by one trained physician in each center
Orouji et al.^58^ Iran 2017	Randomized controlled trial	108	NR	1.8% females, 98.2% males	Educational intervention (counseling sessions)	2 gm nicotine gum	Community-level intervention	Researchers
El Hajj et al.^43^ Qatar 2017	Randomized controlled trial	118 for each group	Majority >18	2.2% females, 97.8% males	The program is based on the trans-theoretical model of change, and tailored behavioral, cognitive and lifestyle strategies	Nicotine replacement therapy: nicotine patch	Implemented in public and private ambulatory, (17) pharmacies in the State of Qatar	Trained pharmacists
Durmaz et al.^37^ Turkey 2019	Randomized controlled trial	132 patients admitted to smoking cessation outpatient clinic	Majority 35–44	39.4% females, 60.6% males	Over the mobile phone intervention (using a messaging application) for smoking cessation and relapse prevention)	NA	Ege University Hospital, Department of Public Health, Smoking Cessation Clinic, between March and July 2017	Healthcare provider (physicians)
Heydari et al.^44^ Iran 2010	Quasi-experimental study	286	42.4 ± 13.4	25.4% females, 74.6% males	‘Cold turkey’ method and cognitive behavioral therapy	Antidepressant treatment (trazodone) and nicotine replacement	Smoking cessation clinic of Iranian National Research Institute of Tuberculosis and Lung Diseases	Physicians and nurses
Heydari^53^ Iran 2017	Quasi-experimental study	227	43.1^§^	41.6% females, 58.4% males	Information and instructions for quitting, 3 visits by the physicians in the first week, and phone call follow-up at 3 and 6 months after abstinence	Champix	Tanaffos smoking cessation clinic in Tehran	Physicians
Shahrokhi et al.^25^ Iran 2008	Cohort study	34154	Majority ≥18	Year 1998 1.8% females, 98.2% males Year 2000 2.2% females, 97.8% males Year 2002 2.6% females, 97.4% males Year 2004 9.1% females, 90.9% males	4 Quit and Win campaigns	NA	Research center	Local sponsors
Heydari et al.^36^ Tehran 2012	Cohort study	308	42.4 ± 13.4	31.5% females, 68.5% males	‘Cold turkey’ method of cessation, cognitive behavioral therapy, educational methods and consultations	4 different types of nicotine replacement therapy (patches, chewing gum, tablets or both patches and gum)	Smoking cessation clinic of the Iranian National Research Institute of Tuberculosis and Lung Diseases	Physicians and nurses
Hawari et al.^47^ Jordan 2012	Cohort study	156 cancer patients	50.3^§^	28.2% females, 71.8% males	NA	Nicotine replacement therapy with varenicline or bupropion	Quit Smoking in Cancer Center Patients in Jordan – smoking cessation clinic	Physicians
Hawari et al.^48^ Jordan 2013	Cohort study	201 cancer patients	49.0^§^	19.9% females, 80.1% males	NA	Nicotine replacement therapy with varenicline or bupropion	King Hussein Cancer Center referred to the smoking cessation clinic Duration of intervention: 1 year	Medication and overall managing are provided by physicians, but counseling by a psychiatrist
Pekel et al.^59^ Turkey 2015	Cohort study	581	NR	NR	Behavioral counseling	Bupropion, varenicline, NRT	Smoking cessation center	NR
Salepci et al.^56^ Turkey 2016	Cohort study	920	42.9 ± 11.5	40.4% females, 59.6% males	NA	Varenicline, bupropion, nicotine patches, nicotine gum	Smoking cessation clinic	Physicians
Turan and Turan^62^ Turkey 2016	Cohort study	179	35.6^§^	7.3% females, 92.7% males	Education about SC benefits, cessation process, possible withdrawal symptoms was provided	NRT, varenicline, and bupropion	Prison	Physicians and researchers
White et al.^49^ Iran 2016	Cohort study	100	40.1 ± 10.9	11% females, 89% males	Psychosocial support, group support	DST nicotine replacement method	Congress 60 (a recovery community in Iran)	Trained guide (achieved at least 3 months of SC)
Marakoğlu et al.^50^ Turkey 2017	Cohort study	3322	37.19 ± 12.02	19.2% females, 80.8% males	Smoking cessation support and behavioral therapy		Selcuk University, School of Medicine, Smoking Cessation Outpatient Clinic	Physicians, researchers
Cetinkaya et al.^41^ Turkey 2018	Cohort study	857	18–65	49.8% females, 50.2% males	All participants received cognitive-behavioral therapy, 12.8% were not prescribed any medical therapy	Varenicline, bupropion, and NR	Smoking cessation clinic in Turkey	Trained physicians
Shoorijeh et al.^38^ Iran 2019	Cohort study	425	52.7 ± 16.3	22.8% females, 77.2% males	Phone consultation	NA	Smoking cessation program for hospital inpatients	Researchers
Esen et al.^61^ Turkey 2020	Cohort study	505 patient files Male: 309 Female: 196	38.9 ± 10.3	38.8% females, 61.2% males	Cognitive behavior therapy is provided by experienced specialists Counseling, and behavioral therapy during the first interview and follow-up	Using varenicline, bupropion, and NRT	Smoking cessation outpatient clinic in Turkey	Physicians
Heydari et al.^52^ Iran 2007	Cross-sectional study	715	38.3 ± 14	20.3% females, 79.7% males	Educational methods, consultation, cognitive-behavioral therapy	Nicotine gum	Smoking cessation clinic	Physicians
Hamadeh et al.^54^ Bahrain 2017	Cross-sectional study	194	37.2 ± 13.9	100% males	Counseling sessions	NRT, nicotine chewing gums and patches combined. Bupropion or champix.	Tobacco clinic only for males in Bahrain	Physicians and staff
Bacha et al.^63^ Lebanon 2018	Crosss-ectional study	156 enrolled patients	≥18 years	48.7% females, 51.3% males	20–30 min of consultation with physiologist	NRT	Enrolled in an outpatient health center in Lebanon for three months	Respiratory physician, nurse, psychologist, dietician
Karadoğan et al.^51^ Turkey 2019	Cross-sectional study	417	44.0 ± 13.7	35% females, 65% males	NA	Varenicline, bupropion, and NRT	Government hospital’s smoking cessation clinic	One pulmonologist (certified for SC counseling by the Turkish Ministry of Health), one nurse, and one medical secretary
Sharifi et al.^55^ Iran 2012	Pre-post study	132	Males: 37.3 ± 10.7 Females: 40.7 ± 12.2	12.9% females, 87.1% males	Counseling sessions	Nicotine gum	Inner-city smoking cessation clinic	Smoking counselors
Öztuna et al.^39^ Turkey 2007	Pre-post study	350	37.4 ± 11.8	42% females, 58% males	Counseling (face-to-face, by phone)	Pharmacotherapy (nicotine replacement therapy)	Smoking cessation clinic	Public Health and Chest Diseases departments

Age of Tobacco Cessation Clinics (TCCs) patients is presented as mean (standard deviation). NA: not applicable. NR: not reported. NRT: nicotine replacement therapy. TTM-based: trans-theoretical model based. DST: a method of gradual treatment or reducing drug use. SC: smoking cessation. TB: tuberculosis.

§ SD not reported.

The age of the participants ranged 18–69 years. The total number of participants in the RCTs was 1694, with the mean age ranging 18–80 years. These studies published between 2006–2019 were reported from Iran, Turkey, Syria, and Qatar. One RCT used a questionnaire to assess the smoking cessation rate. Most RCTs have reported the rate of nicotine dependence using the Fagerström test for nicotine dependence. In the case of the included cross-sectional studies, the sample size ranged from 417 to 1020 smokers, with 2502 participants being in the age range 13–89 years. These studies were published between 2007 and 2019. In the three pre-and-post studies that were included in the present review, the sample size ranged from 36 to 350, with age ranging 37–53 years. These were published in 2007, 2009, and 2012. The sample size of the quasiexperimental studies ranged from 227 to 286, and 513 tobacco smokers were screened. The age of the screened participants ranged 40–56 years, and the studies were published between 2009 and 2017.

### Tobacco cessation programs in the ME

Eighteen studies [cohort (n=6), RCTs (n=5), cross-sectional (n=3), quasi-experimental design (n=2), before-and-after (n=2)] used both behavioral change and pharmaceutical cessation methods for TC. Four studies used only pharmaceutical cessation methods, three of which were cohort studies, and one was a cross-sectional study. Eight studies used only behavioral change cessation methods, four of which were RCTs, three included a cohort, and one was a pre-and-post study. The details are presented in Table 2.

Behavioral change cessation methods included counseling, cognitive behavioral therapy, educational materials, psychological support, physician consultation, motivational interviewing, quitting and winning, and group support. Pharmaceutical cessation methods included nicotine replacement therapy (gum and nicotine patches), varenicline, and bupropion. Five studies included quitlines as a method for behavioral counseling for tobacco cessation in Turkey and Iran, and the methods used were telephone-based counseling^35^, brief phone behavior therapy counseling and education^36^, intervention using the mobile phone (messaging application) for smoking cessation and relapse prevention^37^, phone consultation^38^, face-to-face, and phone counseling^39^.

### Healthcare professionals providing TC programs

Various healthcare professionals provided TC programs. As seen in Table 2, the majority of studies included physicians (n=17), followed by nurses (n=6), researchers (n=6), medical secretaries (n=2), smoking counsellors (n=2), psychologists (n=1), dietitians (n=1), pharmacists (n=1), pulmonologists (n=1), and public health practitioners (n=1). Only two studies reported that physicians were trained to carry out cessation programs^40,41^.

### Outcomes of the TC programs

The effectiveness of TC programs in most of the included studies was evaluated on the basis of the outcomes measured. The outcomes and aims of the included studies are summarized in Table 3. The most commonly reported outcome was the quit rate (n=18)^36,37,40,42-56^, followed by cessation rate (n=8)^39,41,56-61^, sustained abstinence (n=1)^35^, fail to quit (n=1)^62^, cessation survival rate (n=1)^38^, and success rate (n=1)^35^. The majority of studies utilized a self-reported questionnaire to estimate quit rates, while some others used the carbon monoxide breath test to determine quitting state (n=19)^35-37,39-41,43,44,46-48,50-53,^ ^55-57,59^.

**Table 3:** Factors associated with tobacco cessation programs effectiveness in the Middle East

Authors Country Year	Type of study	Aim of the study	Outcome (s) reported (%)	Factors associated with program’s effectiveness
Yilmaz et al.^42^ Turkey 2006	Randomized control trial	To determine if mothers receiving a SC program that focuses on health risks of environmental tobacco smoking (ETS) for their kids have higher quit rate compared to mothers who received SC program that focuses on their own health, or control group mothers.	Quit rate Child intervention: 24.3% Mother intervention: 13% Control: 0.8%	Discussion about the health effects of smoking on children and/or parents Maternal education
Ybarra et al.^35^ Turkey 2012	Randomized control trial	To report cessation rates observed in a messaging-base SC program for adult smokers.	Primary outcome Sustained abstinence at 3 months Intervention group: 11% Control group: 5% Secondary outcome 7- day point prevalence Intervention group: 12% Control group: 9%	Provide in person contact Provide psychological support. Talk about benefits of quitting smoking and its dangers Intervention group females were 4.5 times more likely to quit than control group females (95% CI: 1.2–16.0) Among light smokers, intervention group participants (17%, n=5) were significantly more likely to quit compared to control group participants
Heydari et al.^44^ Iran 2012	Randomized control trial	To evaluate the effectiveness of varenicline for tobacco cessation.	Quit rate 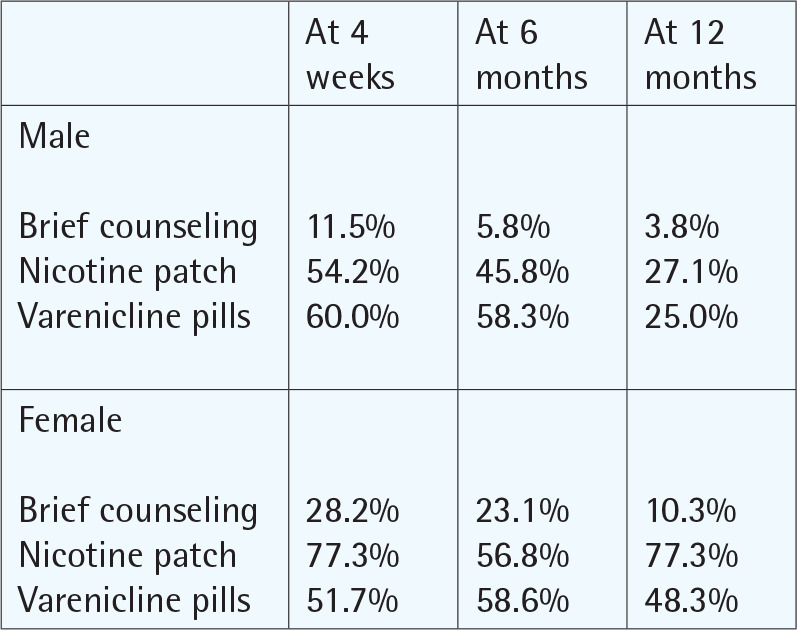	Varenicline has high effectiveness but not highly significantly differs from NRT Free of charge intervention Average nicotine dependence score of 5.5 ± 2.8 (0–10)
Ward et al.^57^ Syria 2013	Randomized control trial	To evaluate nicotine patches and whether they boost smoking cessation rates along with behavioral support in primary health care clinics.	Cessation rates Primary end-point (prolonged) End of treatment: placebo (20.0%), nicotine (21.6%) 6 months: placebo (14.1%), nicotine (13.4%) 12 months: placebo (11.9%), nicotine (12.7%) Secondary end-point (7-day point) End of treatment: placebo (25.9%), nicotine (25.4%) 6 months: placebo (19.3%), nicotine (14.2%) 12 months: placebo (14.8%), nicotine (20.1%)	Patients who experience less withdrawal symptoms had higher likelihood to abstain for long period (p=0.005).
Koyun and Eroğlu^60^ Turkey 2016	Randomized control trial	To determine the influence of transtheoretical model (TTM)-based counseling, training, and a 6-month follow-up on smoking cessation in adult females.	Cessation rate At 6 months follow-up 2.6% in control group 23.7% in intervention group	In the intervention group behavioral processes increased over time (p<0.05), pros of change increased (p<0.05), cons of change decreased (p<0.05), and self-efficacy increased over time This increase in TTM component scores was due to the use of TTM-based counseling and training Cognitive processes are the only component of TTM that was not statistically different in the two groups
Aryanpur et al.^40^ Iran 2016	Randomized control trial	To evaluate the effectiveness of two smoking cessation methods among newly diagnosed pulmonary tuberculosis patients at the clinic.	Quit rate At 6 months combined intervention group: 71.7% brief advice group: 33.9% control group: 9.8%	Medical treatment + behavioral therapy interventions (combined intervention) group and brief advice group had 35 times (p<0.001, OR=35.26, 95% CI: 13.77–90.32) and 7 times (p<0.001, OR=7.14; 95% CI: 2.72–18.72) more odds of not being an active smoker compared to the control group Patient with health problems have higher odds of quitting smoking from brief advice
Orouji et al.^58^ Iran 2017	Randomized control trial	To determine the strength of smoking cessation behavior based on a transtheoretical model.	At 6 months follow-up 40% of intervention group reached maintenance stage of smoking cessation	Cognitive processes, behavioral processes, and smoking temptation showed significant change in the intervention group during 6 months follow-up (p<0.05) Individual counseling, providing the proper amount of nicotine based on dependence level, and helping patients who are willing to quit is important for SC program success
El Hajj et al.^43^ Qatar 2017	Randomized control trial	To test the impact of a structured smoking cessation program delivered by trained ambulatory pharmacists in pharmacies.	Quit rate At 12 months Intervention group: 14.3% Control group: 2.7%	Motivation to quit Free SC services Live healthier 88/152 (57.9%) Religious reasons 55/129 (42.6%) Success to quit Anti-smoking awareness publicly and applying legislative tobacco smoking measures Pharmacists smoking cessation training at HMC The model (HMC) helps to prepare the participant to enter action stage Seeking family support Follow-up sessions even after quitting to help/support Failure to quit High nicotine dependence
Durmaz et al.^37^ Turkey 2019	Randomized control trial	To evaluate the impact of support messages through WhatsApp application added to the usual care of a university hospital cessation unit, compared to usual care alone, on abstinence rates at first 4 weeks.	Quit rate At 1 month 65.9% for the intervention group, and 40.9% for the control group At 3 months 50.0% for the intervention group, and 30.7% for the control group At 6 months 40.9% for the intervention group, and 22.7% for the control group	Follow-up after abstinence and face-to-face follow-up resulted in a significant success to quit smoking Smoking cessation success increased after using the WhatsApp by 1.67% For each increase in the number of quit attempts, the abstinence rate increased by 1.39 times
Heydari et al.^44^ Iran 2010	Quasi-experimental study	To compare the effect of the four types of NRT on the quit rate.	Quite rate At 6 months 38% both genders p=0.08 At 12 months 40% both genders p=0.02	Providing NRTs is positively related with quit rate p=0.000 Nicotine patches found to be about 3 times more effective than other forms of NRT p=0.039
Heydari^53^ Iran 2017	Quasi-experimental study	To evaluate the duration of using Champix (Varenicline) based on its cost.	Quit rate At 1 month 51.1% At 3 months 43.6% At 6 months 20.6%	The use of varenicline for 6 weeks or more, increases the quitting success rate compared with using it for a shorter time significantly p<0.000 The reason of not continuing taking varenicline and the difference in durations was the cost
Shahrokhi et al.^25^ Iran 2008	Quasi-experimental study	To evaluate the effect of a nursing smoking cessation intervention based on the transtheoretical model of change on a sample of military students.	Quit rate At 6 months follow-up 8.3%	The participants’ stages of change before and after the program were significant (p<0.05) The amount smoked per day comparing the time 1, 2, 3, 4 and 5 (t = 5.73, 4.87, 4.27, 4.16, p<0.01) The average cigarettes smoked per day by students showed a statistically significant decrease after the smoking cessation program (Wilks’ lambda = 0.47, p<0.05)
Heydari et al.^36^ Tehran 2012	Cohort study	To assess the efficacy of this Quit and Win contests campaign in the short-term and long-term quitting rates, and also assess some of the factors associated with quitting.	Quit rate At 1 month (self-reported) In 1998 (41.8%), and in 2004 (92.8%) At 1 year (follow-up) In 1998 (22.5%), and in 2004 (91.2%)	Participation in an international competition affected the decision to quit or reduce cigarettes consumption (more than 60% of the participants) In all 4 campaigns, 1 month quit rate and taking the decision to quit at the time of the contest were important indicators for long-term quitting success In 2002 and 2004 campaigns, participants who received advice from health professionals had higher odds to quit by 2.60 (95% CI: 1.30–3.20) and 3.10 (95% CI: 2.50–3.80) than those who did not receive advice The most reported reasons for relapse were withdrawal symptoms (around 59% of who relapsed) and the poor knowledge about how to quit (around 16.4% of those that relapsed)
Hawari et al.^47^ Jordan 2012	Cohort study	To compare quit rates of different formulations of nicotine replacement among clients.	Quit rate After 4 weeks: 88.2% At 6 months: 54.9% At 12 months: 36.2%	Combining nicotine patches and gum had the higher quit rate After 4 weeks: 95.2% At 12 months: 62.5%
Hawari et al.^48^ Jordan 2013	Cohort study	To measure the abstinence rates and identify reasons for the failure to quit smoking in patients visiting a smoking cessation clinic in a comprehensive cancer center.	Quit rate At 12 months was 21.2% Type of treatment and quit rate Bupropion ± NRT: 6% Varenicline ± NRT: 22% NRT: 3% Counseling: 2%	Reasons for success Varenicline ± NRT (22%) 45% patient had smoking-related cancer Reasons for failing to quit Not being able to handle withdrawal symptoms followed by seeing no value in quitting (late stage of cancer); at six months and one year, failure to abstain was most commonly due to facing a stressful personal or professional situation
Pekel et al.^59^ Turkey 2015	Cohort study	To measure the abstinence rates and identify reasons for the failure to quit smoking in patients visiting a smoking cessation clinic in a comprehensive cancer center.	Quit rate At 3 months: 24.4%	Older patients more likely to quit than younger patients (p=0.016) Married patients more likely to quit than single patients (p=0.05) Patients with lower CO levels more likely to quit than those with higher levels (p=0.01)
Salepci et al.^56^ Turkey 2016	Cohort study	To establish the rate of smoking cessation and restarting in one year at the Balçova smoking cessation center.	Cessation rate At 1 year: 30.1% Relapse rate: 51.3%	Patients with high dependence levels were more likely to restart smoking (p<0.001) Relapse was lower in those who had pharmacotherapy than those who had behavioral therapy (p<0.05) The rate of relapse was low in patients who took varenicline (p<0.001)
Turan and Turan^62^ Turkey 2016	Cohort study	To compare smoking cessation rates between patients who had free medications during the period of the project, and those who had to pay for their medication.	Cessation rates At 1 month: Paid:75%, Free:43.5% (p=0.001) At 3 months: Paid:42.2%, Free:25.4% (p=0.002) At 6 months: Paid: 27.3%, Free:14.8% (p=0.008) At 1 year: Paid:18.2%, Free:12.2% (p=0.059)	When quitters at 6 months and 1 year and non-quitters characteristics were compared, age was greater for those who quit (p=0.003) Patients who quit at 6 months have significantly higher Fagerström score, more smoked cigarettes (as pack-years), pathological chest X-rays findings, and paid for their medication (p=0.021, 0.018, 0.013, 0.012)
White et al.^49^ Iran 2016	Cohort study	To assess the smoking-related behaviors and the effectiveness of tobacco cessation therapy in prison.	Fail to quit smoking	Prisoners who have high level of nicotine dependence smoked more cigarettes in prison (p<0.001) High price of tobacco cessation medications (40%), and the environment of prison (35%), were the most common reasons for participants failure to quit
Marakoğlu et al.^50^ Turkey 2017	Cohort study	To explore the introduction of a smoking cessation track within Congress 60 (a prominent recovery community within Iran).	Quit rate During the months of NRT use: 85%	Cessation rates improve with prolonged NRT Peer support groups help in SC
Cetinkaya et al.^41^ Turkey 2018	Cohort study	To compare the smoking cessation rate in the 1st month, 3rd month, 6th month, 1st year, and 2nd year among those who quit smoking after taking different pharmacological and behavioral therapies.	Quit rate At 1 month: varenicline + BT users (63.5%), bupropion + BT users (49.9%), NRT + BT (53.2%), BT (17.1%) At 3 months: varenicline + BT (46.8%), bupropion + BT (35.6%), NRT + BT (24.3%), BT (7.1%) At 6 months: varenicline + BT (34.4%), bupropion + BT (28.4%), NRT (27.3.%) , BT (6.7%) At 2 years: varenicline + BT (19.9%), bupropion + BT (16.0%)	Varenicline + BT success rate in the 12th week was 1.27 times (95% CI: 1.16–1.39, p<0.001) higher than bupropion + BT success rate, and 1.92 times greater (95% CI: 1.08–3.41, p=0.07) than NRT + BT success rate, and 6.55 times (95% CI: 2.81–15.29, p<0.001) greater than behavioral therapy success rate
Shoorijeh et al.^38^ Iran 2019	Cohort study	To know the smoking cessation rate in terms of method used to quit among patients presenting to a smoking cessation clinic in Turkey.	Cessation rate At 1 year: 34.3%	The participant who received a combination of treatments (behavior motivation + counseling + bupropion) recorded the highest success rate among participants Smoking cessation rate is highly correlated with having a chronic condition NRT has higher rate in smoking cessation (47.9%) More men (37.7%) quit smoking than women (30.9%)
Esen et al.^61^ Turkey 2020	Cohort study	To investigate the effects of smoking cessation program on inpatients and factors that may affect success.	Cessation survival rates (CSR) At 1 month: 76% At 2 months: 63% At 3 months: 61% At 4 months onward: 60%	CSR for inpatients who did not smoke at least 30 days before entering cessation program were greater than those of inpatients whose last smoking event was <30 days previously (p=0.05) CSR for inpatients who use more than one item to smoke were higher than those using only cigarettes or hubblebubble (p=0.001) Inpatients who have mild nicotine dependence had greater CSR than Those with sever nicotine dependence (p=0.002) Patients who were interested to stop smoking were more likely to quit (p=0.037)
Heydari et al.^52^ Iran 2007	Cohort study	To investigate smoking cessation rates, the effects of follow-up visits and pharmacological therapies on smoking cessation in smoking cessation clinic in Turkey.	Cessation rate At 1 year: 45.3% Relapse rate: 26.8%	The smoking cessation rate of the users who used varenicline was significantly higher than those who used bupropion (p=0.033) Smoking cessation rate with a Fagerström score <6 was significantly higher than that with a Fagerström score ≥6 (p=0.008) The smoking cessation rate of males (57.9%) was significantly lower than that of females (68.4%) (p=0.019)
Hamadeh et al.^54^ Bahrain 2017	Cross-sectional study	Study the correlation between nicotine dependence rate and outcome of smoking cessation among the entrants of smoking cessation clinic.	Quit rate 65.1%	Rate of success was high among smokers with low nicotine dependence after excluding those who failed to complete the course (n=79; 94%) (p=0.02) Nicotine dependence rate increases by higher number of packs/year consumption (p=0.00)
Bacha et al.^63^ Lebanon 2018	Cross-sectional study	To determine the quit rates among male attendees of quit tobacco clinics (QTC) in Bahrain and describe related factors.	Quit rate 56.5% during study period 37.6% at 6 months or longer after QTC services	More than 3 visits to the clinics along with previous quit attempts of 21 months duration or more were related to success of quitting all types of tobacco (p<0.05)
Karadoğan et al.^51^ Turkey 2019	Cross-sectional study	To assess factors associated with the success rate of smoking cessation among Lebanese smokers in a smoking cessation center.	Success rate 58.9% Failure rate 41%	The number of packs smoked per year decreased the odds of smoking cessation success (OR=0.982: 95% CI: 0.97–0.994 , p =0.004) Moderately and highly motivated smokers had more success in quitting smoking (p=0.027) Patients who were compliant with treatment succeeded quitting smoking (p=0.001)
Sharifi et al.^55^ Iran 2012	Pre-post study	To evaluate the demographic characteristics and other factors that influence the success of smoking cessation among program participants who completed a 5-year follow up.	Cessation rate After 5 years: 34.6%	Participants who used NRT abstained from smoking 1.9 times longer (95% CI: 1.2–2.9) than those not receiving NRT Participants who did not suffer from withdrawal symptoms remained abstinent 2.3 times longer (95% CI: 1.5–3.4) than those with withdrawal symptoms
Öztuna et al.^39^ Turkey 2007	Pre-post study	To evaluate the influence of harm reduction approach in the patterns of smoking of subjects who attended smoking cessation clinic.	Quit rate At 6 months: 12.9%	Number of smoked cigarettes and nicotine gums were 22.9 and 0.03 at the beginning of the study, and after 6 months follow-up the number of smoked cigarettes decreased to 11.3 and the use of nicotine gum increased to 5.63 (p<0.001), which means that harm reduction approach worked and decreased the number of daily cigarettes by approximately 50%.

The table shows type of studies included in the SR, their aims, outcomes in percentage (%), and the factors associated with TC programs effectiveness. TC: tobacco cessation. HMC: Hamad Medical Corporation. NRT: nicotine replacement therapy. CO: carbon monoxide. TTM: trans-theoretical model. BT: behavioral therapy. QTC: Quit Tobacco Clinic. CSR: cessation survival rate. OR: odds ratio. CI: confidence interval

### Factors associated with TC programs’ effectiveness

The various factors associated with the effectiveness of TC programs identified in this review are summarized in Table 3.

*Individual factors*

A number of individual factors are associated with the effectiveness of TC programs. For example, factors found to be associated with reduced effectiveness include individual withdrawal symptoms^39,45,47,57, 63^, high nicotine dependence rate^36,38,43,49,52,60,62,63^, poor knowledge on how to quit^45^, change in attitudes and behaviors^43,56^, stress^47,48^, number of cigarettes smoked per day^62,63^, inability to afford higher priced medications^49,50,62^, and concern about gaining weight^51,63^. Other individual factors that were found to be associated with effective TC programs included older age^51,62^, female gender^61^, knowledge on the benefits of tobacco cessation^35,59^, health issues ^41,47,53,54,59,61-63^, higher education level^48,54^, religious beliefs^59^, and adherence to treatment^51^.

*Interpersonal factors*

We found three interpersonal factors to be associated with TC program effectiveness: family support to quit^45,54^, smoking family member^61^, and peer support groups^40^. The community factors associated with the TC program’s effectiveness include motivational programs such as competition^45^, and psychosocial support^49^.

*Organizational factors*

The organizational factors in our review were represented by the type of services provided in TC programs. Services that were linked to effective TC programs included using NRT^39-41,43,44,47,48,51^, individual counseling^43,54^, combined programs, such as combining NRT with Cognitive Behavioral Thereby (CBT)^41,46,53,58^, follow-up and frequent visits to TC clinics^35,37,51,54,61^, long duration of treatment^41^, applying TTM in designing programs^43,56^, providing advice through health professionals^45^, and use of harm reduction approach in the programs^55^.

*Environmental factors and policy*

The major factors identified at the environmental and policy levels are linked to effective TC programs. These include free-of-charge treatment and cessation programs^36,51,59,63^, and availability of legislative tobacco control measures^59^. The physical environment in which the intervention was carried out plays a crucial role in influencing the effectiveness of quitting smoking; for instance, smokers in prison showed substantially lower success rates^49^.

### Quality assessment

The results of the quality assessment are presented in Supplementary file Tables 1 to 5. According to the Cochrane risk of bias assessment tool for RCTs, only one study had a low risk of bias, while five raised some concerns of bias, and three were at a high risk of bias. Based on MINORS, for quasi-experimental studies, one comparative study scored 17 and did not reach the global ideal score for comparative studies, whereas the other two non-comparative studies scored 16 or above, which is within the global ideal score for non-comparative studies. The quality assessment tool for cross-sectional studies revealed that the majority of studies had fair to good quality since most domains constituting an integral aspect were met.

For the pre-and-post studies, the quality assessment tool showed that the blinding of outcome assessor and follow-up were not applicable; thus, the outcome may not have been accurately measured, deeming the quality of the two studies fair to poor. When the cohort studies were assessed using the CASP tool, it was noted that either most of the studies did not identify or account for confounding variables or the variables were not precise; thus, the results cannot be generalized to the local population, making the quality of these studies fair to poor.

## DISCUSSION

### Summary of findings

The present systematic review was performed to identify and explore the various TC programs implemented in the ME and assess the factors that influence the effectiveness of these programs. Thirty articles in ME met the inclusion criteria. The majority of the included studies employed a cohort study design, and the other 20 studies were RCTs, crosssectional, quasi-experimental, and pre-and-post. The vast majority of studies had implemented programs that integrate both pharmacological and behavioral TC interventions. While some reported that different HCPs facilitated these programs, including nurses, physicians, dietitians, medical secretaries, smoking counsellors, psychologists, pharmacists, pulmonologists, and public health practitioners, very few reported provision of training for the uptake of these programs. Meanwhile, the PHS guidelines state that the provision of more interventions and the wider the diversity of healthcare providers delivering these interventions, the more likely is that individuals will effectively quit smoking and remain abstinent^64^. It is imperative to emphasize the need for provider training, as this will have an additive influence on patients’ outcomes with regard to smoking cessation^65^.

Our systematic review identified different factors, including individual and behavioral, interpersonal, community, organizational, and environmental factors that influenced abstinence rates in TC programs. These findings provide evidence to help develop effective TC programs that encompass these factors, to improve the TC rate in the ME, as well as to support healthcare providers in planning feasible, effective, and culturally appropriate TC programs, and understand the influential factors related to the high abstinence rate in different contexts.

### Factors associated with the effectiveness of TC programs

Through this review, it was clear that individual factors were associated with the effectiveness of TC programs and the success of abstinence. For example, nicotine dependence, which is regarded as an individual factor, causes various changes in the brain, making individuals feel the urge to use the substance, as a result, the appearance of a nicotine withdrawal syndrome after discontinuation increases, making quitting more difficult^66^. To improve the quitting success rate, healthcare professionals need to support patients by offering them behavioral treatments to control their withdrawal symptoms and overcome other obstacles. Moreover, providing insurance coverage to the population for pharmacotherapy and behavioral cessation treatments may have a significant impact on smoking cessation of individuals^67^. Other individual factors, such as education level, are positively associated with abstinence rates, implying that smokers with a higher level of education are more likely to quit smoking and remain abstinent^47^.

In addition, some studies in the ME, including Qatar and Iraq, revealed that there is a lack of knowledge regarding the dangers of tobacco smoking, necessitating the need to raise awareness about this issue^68,69^. The fact that a patient is concerned about being unwell or developing a health problem is a powerful individual factor associated with smoking cessation. As a result, it is worthwhile to concentrate on interventions to raise awareness about the health effects of smoking to increase the quitting rates.

A major finding of five studies in Turkey, Iran, and Bahrain, was that family pressure or support can influence the success of TC. Moreover, peer pressure ranked fourth as a reason why study participants started smoking in the first place^70^. On the other hand, both family members and friends have the power to positively influence and support others in quitting smoking when engaged in TC sessions^71^. There is evidence in the literature that successful smoking cessation is strongly associated with the absence of household members, co-workers, friends, or partners, who smoke^72,73^.

Motivational factors in community programs are essential to quitting smoking^74^. Though research on motivational factors predicts attempts to quit, it does not affect the maintenance of TC. Moreover, earlier findings have shown that motivation is not all that a smoker needs to quit^74^. The use of a community-wide program to provide reinforcement, support, and standards, for not smoking, is an important aspect of health-promotion activities. Additionally, individuals who live with mental health issues, such as depression or schizophrenia, are more likely to be smokers compared to healthy people, and are less likely to stop smoking or maintain a smoke-free lifestyle^75^. This suggests that these people require psychosocial support not only to enhance their psychological health, but also to improve health behaviors such as quitting smoking which could enhance their quality of life^76^. For instance, one study documented a significant reduction in the number of years in individuals with schizophrenia as a result of smoking-related diseases^77^. Competitions are rare in TC interventions but can be applied to support patients in the community through their journey to maintain abstinence.

Our review reported that different factors are associated with abstinence rate when it comes to services and organizational factors that were presented in TC programs. These factors can be divided into two main divisions: one being the type of treatment or therapy provided and the other healthcare providers’ training and education. Most of the programs included services such as individual counseling or CBT, NRTs, a combination of both (NRT + CBT), using harm reduction approach by professionals in the intervention, frequent follow-up in the TC clinic to stay focused on the target and help in maintenance, the application of TTM in the intervention construction, ‘cold turkey’ method, and long duration of treatment^17^. To enhance with evidence, we found that the combination of two different methods, such as pharmacological therapy (e.g. use of varenicline) and CBT (e.g. individual counseling), resulted in a higher score of abstinence^78^. For instance, according to a study conducted in Iran, the abstinence rate after 6 months in the combined intervention group was significantly higher than that in the brief advice group (71.7% vs 33.95%, p<0.001)^40^. Moreover, a small number of studies reported that using a quitline as a method for tobacco cessation and was only mentioned in behavioral therapies rather than pharmacotherapy or both treatments combined. In many outpatient settings, quitline services are linked to noticeably better smoking cessation efforts. Findings of an article show that integrating quitline programs within a specialist preoperative clinic may help cessation efforts^79^. Therefore, it is recommended to develop quitline services and interventions in the ME for better tobacco cessation outcomes^79^.

Physicians must play a role in improving the success rate of smoking cessation by assisting patients and providing directive instructions on management of withdrawal symptoms using several professional approaches such as counseling, interviews, discussions, and other productive methods^80^. In support, findings of a study done in Qatar revealed that 84.8% of respondents believed that receiving cessation advice from a healthcare expert improved the patient’s chances of stopping smoking^81^. In addition, trained physicians may be able to influence the views of tobacco smokers, which can increase the number of patients who seek to stop smoking and remain abstinent. It is imperative to note that trained HCPs performing tobacco cessation tasks, had a significant effect on the point prevalence of smoking, continuous abstinence, and professional performance when compared to non-trained HCPs^82^. Such responsibilities include creating follow-up schedules, requesting patients to set a quit date, counseling smokers’ prescriptions of a quit date, and providing self-help materials. Several health organizations throughout the world have adopted the 5As (Ask, Advise, Assess, Assist, and Arrange) smoking cessation intervention strategy, which was advocated by evidence-based smoking cessation guidelines^8,9^. This model is built to identify the appropriate cessation intervention based on willingness of smokers to quit smoking^83^. According to one study, HCPs using the 5As model had a better positive experience and felt competent in helping others to quit smoking, however, some aspects might need special training in order to be implemented for a successful tobacco cessation^84^.

Our review found that the presence of free-of-charge TC programs and treatments can benefit a number of individuals from various socioeconomic backgrounds, which echoes the findings from another study^85^. In addition to the implementation of tobacco control measures, the presence of smoke-free zones, ban on tobacco advertising, promotion, and sponsorship, sales restrictions, prohibited e-cigarette sales, and tobacco packaging and labeling, can influence the effectiveness of TC programs. According to the findings of one study, comprehensive smoke-free legislation can have a significant impact on quitting attempts^86^. Tobacco-free environmental policies, such as smoke-free areas and smoking bans and restrictions, will undoubtedly improve the effectiveness of TC programs and aid smoking cessation^87^. That is, developing an environment that supports abstinence and provides various types of assistance and encouragement for a wide range of smoking individuals, will eventually create and maintain a tobacco-free culture^87^.

Finally, the physical environment in which the intervention was conducted also had an impact on the program’s effectiveness. According to one study, the physical environment has a significant impact on patient abstinence rate. A study that was conducted in a Turkish prison with 38 participants, showed that 35% of those who failed to quit smoking agreed that the reason for their failed attempt was the inappropriate prison environment^62^. In addition, smoke-free policies are part of the physical environment factors that impact tobacco use. Private and public sector regulations that forbid smoking in indoor workspaces and in specified public areas, are both examples of smoke-free policies. Private sector smoke-free regulations may explicitly prohibit tobacco use on workplace property or limit it to specific outside areas. Community smoke-free policies set smoke-free requirements for all or specific indoor public spaces and industries^88^. According to Hopkins et al.^88^, a systematic review concluded that there is sufficient evidence to support that smoke-free policies implemented reduce tobacco use in workplaces and communities^87^. In addition, evidence from another systematic review shows that smoke-free policies are the best possible way to protect non-smokers from exposure to secondhand smoke^89^.

### Strengths and limitations

To the best of our knowledge, this systematic review is the first of its kind to investigate TC programs and the factors associated with their effectiveness in ME. This review included studies with 45764 participants, which may enable generalizing the findings to the population of the ME region. Moreover, the review addresses the factors that are associated with the effectiveness of TC programs for both sexes and across different age groups. The majority of research conducted on this topic in the ME is in the form of cohort studies and RCTs, which are ranked highest in the hierarchy of evidence. Cohort studies are distinguished by their ability to measure all variables of interest and the ease with which large samples can be obtained. Likewise, RCTs are known for their ability to minimize the risk of confounding and provide the most reliable research design.

Nevertheless, the limitations of our systematic review need to be mentioned. First, the lack of data for some countries due to cultural reasons (women not smoking or underreporting of smoking status of women due to stigma) may limit the generalizability of findings to ME countries^90^. Second, any bias that arises as a result of publication may have an impact on our findings. Third, although our systematic review included studies from many different countries throughout the ME, not all factors could be applied equally because of heterogeneity in terms of context, environment, and culture. Heterogeneity can also be found in healthcare systems and in the availability of resources across countries, which leads to the inability to generalize the results across countries. Fourth, the included studies were of fair to poor quality according to the risk of bias assessment, emphasizing the need for conducting high-quality studies following certain guidelines based on study type for more accurate findings in the ME region. Finally, one disadvantage of RCTs is that they use self-administered questionnaires, which makes it difficult to establish causal relationships.

### Implications

The findings of our review reflect gaps in the TC literature. For a comprehensive assessment of the factors affecting the effectiveness of cessation programs, future research should focus on examining the sociocultural and economic factors that might influence these programs. In addition, more research is needed to understand the barriers to seeking TC services in the ME and tackling these barriers to enhance smoking cessation in both genders. The findings also highlight the importance to conduct high-quality studies, since most of the included studies in our review were of average to poor quality according to the risk of bias assessment.

In terms of practice, our review suggests that multiple factors, including individual, interpersonal, organizational, and policy, should be considered for effective TC programs in the ME. The findings revealed that the most effective forms of cessation services were those that employed a combination of methods (pharmaceutical and behavioral change therapies). Providers of cessation programs should be trained and equipped with counseling skills to assist patients in quitting smoking, as our review revealed that trained providers had a significant positive impact on the rates of quitting smoking and abstinence. Moreover, it is equally important to examine the factors that prohibit individuals from quitting smoking while constructing or designing any TC program. Finally, establishing cost-free smoking cessation programs could help encourage more patients to quit smoking in many countries in the ME. In addition, among other services that could encourage smoking individuals to quit include visible access to smoking cessation such as using virtual counseling or mobile smoking cessation clinics, and free delivery of smoking cessation medications. Long-term follow-up studies (longitudinal and cohort research designs) with young participants are also suggested in order to assess the maintenance of tobacco cessation, by adhering to the reporting guidelines for the conducted study design so that researchers will have simple access to the crucial information.

## CONCLUSIONS

The present systematic review aids in exploring different TC services and identifying the various factors that contribute to the effectiveness of TC programs implemented in the ME. Understanding these factors and how they are embedded at different levels would support healthcare providers in planning evidence-based and multilevel effective TC programs to achieve high rates of abstinence. The current findings imply the need for more focused interventions performed by trained HCPs in the ME for effective tobacco cessation. It is also advised to take into account quitline services due to their accessibility and convenience for smokers.

## Data Availability

The data supporting this research are available from the authors on reasonable request.
