# Supplementary material for: Tobacco cessation programs and factors associated with their effectiveness in the Middle East: A systematic review
Source: Tob Induc Dis. 2022 Nov 3;20:84. doi: 10.18332/tid/153972 (PMC9631871; doi:10.18332/tid/153972)
Supplement: Supplementary file 1 [file TID-20-93-s1.pdf]

SUPPLEMENTARY TABLES

Supplementary Table 1. Cochrane Risk of Bias Tool for assessing the quality of Randomized Controlled Trails

Author Name, Country—Year of Publication	Domain 1	Domain 2	Domain 3	Domain 4	Domain 5	Domain 6	Domain 7
1. G. Yilmaz, Turkey—2006 [1]	Unclear	Low	Low	Unclear	Unclear	Low	Low
2. Michele Ybarra, Turkey—2012 [2]	Low	Low	Low	Unclear	High	Unclear	Low
3. Heydari, G., Iran—2012 [3]	Low	High	Low	Unclear	Unclear	Unclear	Low
4. Kenneth D. Ward, Syria—2013 [4]	Low	Low	Low	Low	Low	Low	Low
5. Ayşe KOYUN, Turkey—2016 [5]	Low	High	Low	Low	Unclear	Unclear	Low
6. Aryanpur, M.,Tehran—2016 [6]	Low	Unclear	Low	Unclear	Unclear	Low	Low
7. Mohammad Ali Orouji, Iran—2017 [7]	Low	Unclear	Low	Unclear	Unclear	Unclear	Low
8. Maguy Saffouh El Hajj, Qatar—2017 [8]	Low	Low	Low	Unclear	Low	Low	Low
9. Durmaz,S., Turkey—2019 [9]	Low	Unclear	Low	Unclear	Unclear	Low	Low

The five domains are: selection, performance, attrition, reporting, and other. The overall risk of bias judgment is either low risk of bias (All domains are low risk of bias), raises some concerns (at least one domain is unclear risk of bias, but not high risk of bias), or high risk of bias (at least one domain is high risk of bias or there are multiple domains are not clear).

Supplementary Table 2. Methodological Index for Non-Randomized Studies for assessing the quality of Quasi-Experimental studies

**Author Name, Country—Year of Publication**	**A clearly stated aim**	**Inclusion of consecutive patients**	**Prospective collection of data**	**Endpoints appropriate to the aim of the study**	**Unbiased assessment of the study endpoint**	**Follow-up period appropriate to the aim of the study**	**Loss to follow up less than 5%**	**Prospective calculation of the study size**	**An adequate control group**	**Contemporary groups**	**Baseline equivalence of groups**	**Adequate statistical analyses**
S. Ergul, Turkey—2009 (non-comparative)	2 points	2 points	2 points	2 points	0 points	2 points	2 points	2 points	0 points	0 point	0 points	2 points
Gholamreza Heydari, Iran—2010 [10] (comparative)	2 points	2 points	2 points	2 points	0 points	2 points	2 points	1 point	1 point	1 point	1 point	1 point
Gholamreza Heydari, Iran—2017 [11] (non-comparative)	2 points	2 points	2 points	2 points	0 points	2 points	1 point	1 point	1 point	1 point	2 points	1 point

12 items, scored 0 (for not reported), 1 (for reported but inadequate), or 2 (for reported and adequate). For non-comparative studies the global ideal score is 16 or above, and is 24 for comparative studies

Supplementary Table 3. Quality Assessment Tool for Cross-Sectional Studies

**Author Name, Country—Year of Publication**	Gholam Reza Heydari, Iran—2007 [24]	Randah Ribhi Hamadeh, Bahrain—2017 [25]	Bacha, Z. A., Lebanon—2018 [26]	Dilek Karadoğan, Turkey—2019 [27]
1. Was the research question or objective in this paper clearly stated?	Yes	Yes	Yes	Yes
2. Was the study population clearly specified and defined?	Yes	Yes	Yes	Yes
3. Was the participation rate of eligible persons at least 50%?	Yes	Yes	CD	Yes
4. Were all the subjects selected or recruited from the same or similar populations (including the same time period)? Were inclusion and exclusion criteria for being in the study prespecified and applied uniformly to all participants?	Yes	Yes	Yes	Yes
5. Was a sample size justification, power description, or variance and effect estimates provided?	No	No	No	No
6. For the analyses in this paper, were the exposure(s) of interest measured prior to the outcome(s) being measured?	Yes	Yes	Yes	Yes
7. Was the timeframe sufficient so that one could reasonably expect to see an association between exposure and outcome if it existed?	No	No	no	no
8. For exposures that can vary in amount or level, did the study examine different levels of the exposure as related to the outcome (e.g., categories of exposure, or exposure measured as continuous variable)?	Yes	Yes	Yes	Yes
9. Were the exposure measures (independent variables) clearly defined, valid, reliable, and implemented consistently across all study participants?	Yes	Yes	Yes	Yes
10. Was the exposure(s) assessed more than once over time?	No	No	No	Yes
11. Were the outcome measures (dependent variables) clearly defined, valid, reliable, and implemented consistently across all study participants?	Yes	Yes	Yes	Yes
12. Were the outcome assessors blinded to the exposure status of participants?	NA	NA	NA	No
13. Was loss to follow-up after baseline 20% or less?	NA	NA	NA	NA
14. Were key potential confounding variables measured and adjusted statistically for their impact on the relationship between exposure(s) and outcome(s)?	No	No	No	Yes

The tool have 14 criteria, answered with yes, no, or other. Abbreviations: CD: cannot determine. NA: not applicable

Supplementary Table 4. Quality Assessment Tool for Pre-post Studies

**Author Name, Country—Year of Publication**	Funda öztuna, Turkey—2007 [30]	S. Ergul, Turkey—2009 [28]	Hooman Sharifi, Iran—2012 [29]
1. Was the study question or objective clearly stated?	Yes	Yes	Yes
2. Were eligibility/selection criteria for the study population prespecified and clearly described?	yes	Yes	Yes
3. Were the participants in the study representative of those who would be eligible for the test/service/intervention in the general or clinical population of interest?	Yes	Yes	Yes
4. Were all eligible participants that met the prespecified entry criteria enrolled?	Yes	Yes	Yes
5. Was the sample size sufficiently large to provide confidence in the findings?	Yes	No	Yes
6. Was the test/service/intervention clearly described and delivered consistently across the study population?	Yes	Yes	Yes
7. Were the outcome measures prespecified, clearly defined, valid, reliable, and assessed consistently across all study participants?	Yes	Yes	Yes
8. Were the people assessing the outcomes blinded to the participants' exposures/interventions?	NA	NA	NA
9. Was the loss to follow-up after baseline 20% or less? Were those lost to follow-up accounted for in the analysis?	NA	NA	No
10. Did the statistical methods examine changes in outcome measures from before to after the intervention? Were statistical tests done that provided p values for the pre-to-post changes?	Yes	Yes	Yes
11. Were outcome measures of interest taken multiple times before the intervention and multiple times after the intervention (i.e., did they use an interrupted time-series design)?	No	No	No
12. If the intervention was conducted at a group level (e.g., a whole hospital, a community, etc.) did the statistical analysis take into account the use of individual-level data to determine effects at the group level?	Yes	Yes	Yes

The tool have 12 criteria, answered with yes, no, or other. Abbreviations: CD: cannot determine. NA: not applicable

Supplementary Table 5. Critical Appraisal Skills Programme checklist for assessing the quality of Cohort Studies

**Author Name, Country—Year of Publication**	S. Shahrokhi, Iran—2008 [12]	G. Heydari, Tehran—2012 [13]	Hawari F, Jordan—2012 [14]	Hawari F, Jordan—2013 [15]	Özlem PEKEL, Turkey—2015 [16]	Banu Salepci, Turkey—2016 [17]	Onur Turan, Turkey—2016 [18]	William L. White, Iran—2016 [19]	Kamile Marakoglu, Turkey—2017 [20]	Cetinkaya, P. D., Turkey—2018 [21]	Fatemeh Tabatabai Shoorijeh, Iran—2019 [22]	Esen, A., Turkey—2020 [23]
Did the study address a clearly focused issue?	Yes	Yes	Yes	Yes	Yes	Yes	Yes	Yes	Yes	Yes	Yes	Yes
Was the cohort recruited in an acceptable way?	Yes	Can't tell	Yes	Yes	Yes	Yes	Yes	Yes	Yes	Yes	Yes	Yes
Was the exposure accurately measured to minimise bias?	Yes	Yes	Yes	Yes	Yes	Yes	Yes	Yes	Yes	Yes	Yes	Yes
Was the outcome accurately measured to minimise bias?	Yes	Yes	Can't tell	Yes	Yes	Yes	Yes	Yes	Yes	Yes	Yes	No
Have the authors identified all important confounding factors?	Can't tell	Can't tell	No	No	No	Can't tell	No	No	No	No	Yes	No
Have they taken account of the confounding factors in the design and/or analysis?	No	No	No	Can't tell	Can't tell	Yes	No	No	No	No	No	No
Was the follow up of subjects complete enough?	Yes	Yes	Yes	Yes	Yes	Yes	Can't tell	Yes	Yes	Yes	Yes	Yes
Was the follow up of subjects long enough?	Yes	Yes	Yes	Yes	Yes	Yes	Yes	Yes	Yes	Yes	Yes	Yes
What are the results of this study?	Abstinence rate	Abstinence rate	Abstinence rate	3-month abstinence	1-year smoking cessation rate	Smoking cessation rate	Rate of smoking	Abstinence rate	Smoking cessation rate	Smoking cessation rate	Cessation survival rate	Smoking cessation rate
How precise are the results?	Precise (95% CI used)	Can't tell (no 95% CI given)	Can't tell (no 95% CI given)	Can't tell (no 95% CI given)	Precise (95% CI used)	Can't tell (no 95% CI given)	Can't tell (no 95% CI given)	Can't tell (no 95% CI given)	Precise (95% CI used)	Can't tell (no 95% CI given)	Precise (95% CI used)	Can't tell (no 95% CI given)
Do you believe the results?	Yes	Yes	Yes	Yes	Yes	Yes	Yes	Yes	Yes	Yes	Yes	Yes
Can the results be applied to the local population?	Yes	Yes	No	Can't tell	Yes	No	Can't tell	No	Yes	Yes	No	Can't tell
Do the results of this study fit with other available evidence?	Yes	Yes	Yes	Can't tell	Yes	Yes	Yes	Yes	Yes	Yes	Yes	Yes
What are the implications of this study for practice?	Can't tell	Yes	No	No	Yes	Can't tell	Yes	Yes	No	Yes	Yes	Yes

1. Yilmaz G, Karacan C, Yöney A, Yilmaz T. Brief intervention on maternal smoking: a randomized controlled trial. Child Care Health Dev. 2006;32(1):73-9.
2. Ybarra M, Bağci Bosi AT, Korchmaros J, Emri S. A text messaging-based smoking cessation program for adult smokers: randomized controlled trial. Journal of medical Internet research. 2012;14(6):e172.
3. Heydari G, Talischi F, Tafti SF, Masjedi MR. Quitting smoking with varenicline: parallel, randomised efficacy trial in Iran. International Journal of Tuberculosis and Lung Disease. 2012;16(2):268-72.
4. Ward KD, Asfar T, Al Ali R, Rastam S, Weg MW, Eissenberg T, et al. Randomized trial of the effectiveness of combined behavioral/pharmacological smoking cessation treatment in Syrian primary care clinics. Addiction (Abingdon, England). 2013;108(2):394-403.
5. Koyun A, Eroğlu K. The effect of transtheoretical model-based individual counseling, training, and a 6-month follow-up on smoking cessation in adult women: A randomized controlled trial. Turkish Journal of Medical Sciences. 2016;46(1):105-11.
6. Aryanpur M, Hosseini M, Masjedi MR, Mortaz E, Tabarsi P, Soori H, et al. A randomized controlled trial of smoking cessation methods in patients newly-diagnosed with pulmonary tuberculosis. BMC Infectious Diseases. 2016;16(1).
7. Orouji MA, Shojaeizadeh D, Sadeghi R, Rafiei M. Effects of a theory-driven educational package and social support on durability of cigarette smoking cessation behavior: A community-based program. Electron Physician. 2017;9(9):5331-8.
8. El Hajj MS, Kheir N, Al Mulla AM, Shami R, Fanous N, Mahfoud ZR. Effectiveness of a pharmacist-delivered smoking cessation program in the State of Qatar: a randomized controlled trial. BMC Public Health. 2017;17(1):215.
9. Durmaz S, Ergin I, Durusoy R, Hassoy H, Caliskan A, Okyay P. WhatsApp embedded in routine service delivery for smoking cessation: effects on abstinence rates in a randomized controlled study. BMC public health. 2019;19(1):387.
10. Heydari G, Marashian M, Emami H. Efficacy of nicotine patch in combination with Trazodone in smoking cessation. Tanaffos. 2010;9(3):50-7.
11. Heydari G. Is cost of medication for quit smoking important for smokers, experience of using champix in Iranian smoking cessation program 2016. International Journal of Preventive Medicine. 2017;8.
12. Shahrokhi S, Kelishadi R, Sarrafzadegan N, Khosravi A, Roohafza HR, Pooya A, et al. Evaluation of the quit and win contest for smoking cessation in the Islamic Republic of Iran. Eastern Mediterranean Health Journal. 2008;14(6):1270-9.

13. Heydari G, Marashian M, Ebn Ahmady A, Masjedi M, Lando HA. Which form of nicotine replacement therapy is more effective for quitting smoking? a study in tehran, islamic republic of Iran. Eastern Mediterranean Health Journal. 2012;18(10):1005-10.

14. Hawari F, Obeidat N, Beano H, Dawahrah S, Al-Rimawi D, Ghonimat I. Smoking Abstinence Rates and Reasons for Failure to Quit Smoking in Cancer Patients in Jordan. Respiration. 2012;83(3):233-8.

15. Hawari FI, Obeidat NA, Ayub HS, Dawahrah SS, Hawari SF. Smoking Cessation Treatment and Outcomes in Medium to Heavy Cigarette Smokers being Treated for Cancer in Jordan. Asian Pacific Journal of Cancer Prevention. 2013;14(11):6875-81.

16. Pekel Ö, Ergör G, Günay T, Baydur H, Choussein B, Budak R, et al. Smoking cessation and the effect of nicotine dependence on relapse rate in Izmir, Turkey. Turk J Med Sci. 2015;45(4):895-901.

17. Salepci B, Fidan A, Çağlayan B, Parmaksız ET, Kıral N, Cömert SŞ, et al. Can a computer-based prescription of free medication increase smoking cessation rates efficiently? Turk Toraks Dergisi. 2016;17(1):15-21.

18. Turan O, Turan PA. Smoking-related behaviors and effectiveness of smoking cessation therapy among prisoners and prison staff. Respiratory Care. 2016;61(4):434-8.

19. White WL, Daneshmand R, Funk R, Dezhakam H. A Pilot Study of Smoking Cessation within an Iranian Addiction Recovery Community. Alcoholism Treatment Quarterly. 2016;34(1):15-29.

20. Marakoglu K, Kargin NC, Ucar RM, Kizmaz M. Evaluation of pharmacologic therapies accompanied by behavioural therapy on smoking cessation success: a prospective cohort study in Turkey. Psychiatry and Clinical Psychopharmacology. 2017;27(3):227-33.

21. Cetinkaya PD, Turan A, Zerman N, Cetinkaya F, Anar C. SMOKING CESSATION RATES BY METHOD USED TO QUIT AT ONE YEAR AMONG PATIENTS ATTENDING A SMOKING CESSATION CLINIC IN TURKEY. Southeast Asian Journal of Tropical Medicine and Public Health. 2018;49(5):879-86.

22. Shoorijeh FT, Palenik CJ, Askarian M. Effect of a smoking cessation program on inpatients in the largest hospital in Southern Iran. International Journal of Preventive Medicine. 2019;10.

23. Esen A, Soylem Y, Arica S, Belgin G, Gonultas N. Factors affecting success and abstinence within a smoking cessation clinic: A one-year follow-up study in Turkey. Tobacco Prevention & Cessation. 2020;6.

24. Heydari GR, Ariyanpour M, Kashani BS, Ramezankhani A, Tafti SF, Hosseini M, et al. Tobacco Dependency Evaluation with Fagerstrom Test among the Entrants of Smoking Cessation Clinic. Tanaffos. 2007;6(4):47-52.

25. Hamadeh RR, Ahmed J, Al-Kawari M, Bucheeri S. Quit tobacco clinics in Bahrain: smoking cessation rates and patient satisfaction. Tobacco Induced Diseases. 2017;15.

26. Bacha ZA, Layoun N, Khayat G, Allit S. Factors associated with smoking cessation success in Lebanon. Pharmacy Practice-Granada. 2018;16(1).

27. Karadogan D, Onal O, Kanbay Y. How does reimbursement status affect smoking cessation interventions? A real-life experience from the Eastern Black Sea region of Turkey. Tobacco Induced Diseases. 2019;17.

28. Ergul S, Temel AB. The effects of a nursing smoking cessation intervention on military students in Turkey. International Nursing Review. 2009;56(1):102-8.

29. Sharifi H, Kharaghani R, Emami H, Hessami Z, Masjedi MR. Efficacy of Harm Reduction Programs among Patients of a Smoking Cessation Clinic in Tehran, Iran. Archives of Iranian Medicine. 2012;15(5):283-9.

30. Öztuna F, Çan G, Özlü T. Five-year outcomes for a smoking cessation clinic. Respirology. 2007;12(6):911-5.

Appendix A.

Quality assessment

For the Cochrane risk of bias assessment tool for RCTs, high, low, or unclear risk of bias judgments were used to assess the individual elements of the five domains: selection, performance, attrition, reporting, and others. The overall risk of bias judgment is either low (all domains have a low risk of bias), raises some concerns (at least one domain is an unclear risk of bias, but not a high risk of bias), or high (at least one domain is a high risk of bias or multiple domains are not clear) [31]. The MINORS tool consists of 12 items scored as 0 (if not reported), 1 (if reported but inadequate), or 2 (if reported and adequate). For non-comparative studies, the global ideal score is 16 or above, and 24 for comparative studies [32]. The quality assessment tool for cross-sectional studies consists of 14 criteria answered by yes, no, or other (cannot determine, not applicable, or not reported). The overall quality is determined by the authors [114]. The quality assessment tool for before-and-after studies consists of 12 criteria also answered by yes, no, or other (cannot determine, not applicable, not reported); the overall quality being determined by the authors [114]. The critical appraisal skills program for cohort studies consists of three main sections that assess whether the results of the study are valid, what the results are, and whether the results help locally by 12 questions that are answered with yes, no, or cannot tell [34]. If the answers to the first two questions are yes, the authors proceeded with the remaining questions, and the overall quality is determined by the authors

© 2022 Al-Qashoti M.R. et al.
